# The Roles of Autophagy in Cancer

**DOI:** 10.3390/ijms19113466

**Published:** 2018-11-05

**Authors:** Chul Won Yun, Sang Hun Lee

**Affiliations:** 1 Medical Science Research Institute, Soonchunhyang University Seoul Hospital, Seoul 04401, Korea; skydbs113@naver.com; 2 Department of Biochemistry, Soonchunhyang University College of Medicine, Cheonan 31538, Korea

**Keywords:** autophagy, cancer, cancer stem-cells, autophagy modulators

## Abstract

Autophagy is an intracellular degradative process that occurs under several stressful conditions, including organelle damage, the presence of abnormal proteins, and nutrient deprivation. The mechanism of autophagy initiates the formation of autophagosomes that capture degraded components and then fuse with lysosomes to recycle these components. The modulation of autophagy plays dual roles in tumor suppression and promotion in many cancers. In addition, autophagy regulates the properties of cancer stem-cells by contributing to the maintenance of stemness, the induction of recurrence, and the development of resistance to anticancer reagents. Although some autophagy modulators, such as rapamycin and chloroquine, are used to regulate autophagy in anticancer therapy, since this process also plays roles in both tumor suppression and promotion, the precise mechanism of autophagy in cancer requires further study. In this review, we will summarize the mechanism of autophagy under stressful conditions and its roles in tumor suppression and promotion in cancer and in cancer stem-cells. Furthermore, we discuss how autophagy is a promising potential therapeutic target in cancer treatment.

## 1. Introduction

**Figure 1:**
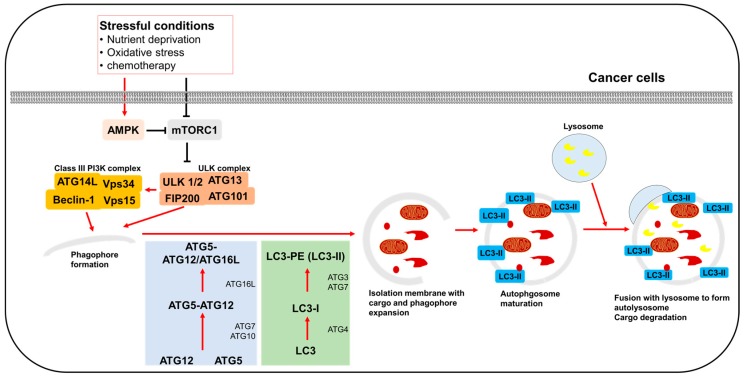
A schema illustrating the regulation of the autophagic pathway under diverse stressful conditions in cancer cells.

Autophagy is a physiological cellular process for the degradation and elimination of misfolded proteins and damaged organelles that functions in adaptation to starvation, development, cell death, and tumor suppression [1,2]. One of the important mechanisms of autophagy is an intracellular degradation pathway mediated by double membrane vesicles called autophagosomes. These autophagosomes deliver degraded cytoplasmic components to the lysosome to be recycled during stressful conditions. This mechanism of autophagy is essential for protecting cells from damaged proteins, to shield cell organelles from toxins, to maintain cell metabolism and energy homeostasis, and to promote cell survival (Figure 1).

Autophagy can be general (non-selective) or selective. General autophagy packages portions of the cytoplasm into autophagosomes and delivers them to lysosomes for degradation. In contrast, selective autophagy works by recognizing specific targets, such as damaged cell organelles, protein aggregates, and intracellular pathogens. Recently, it has been reported that defects of autophagy are associated with genomic damage, metabolic stress, and tumorigenesis [3]. In addition, many studies suggest that autophagy has been linked to both cancer initiation and cancer therapy for several years [4,5]. Indeed, some studies suggest that autophagy is a regulator of many oncogenes and tumor suppressor genes [6,7], whereas other studies have shown that autophagy is involved in both the promotion of tumorigenesis and the development and inhibition of cancer [8,9,10,11].

In this review, we summarize the typical biological mechanism of autophagy, as well as the role of autophagy in cancer, such as in tumor suppression and promotion, cancer-drug resistance, and metastasis. Next, we discuss the interaction between autophagy and cancer stem-cells. Finally, we discuss autophagy as a therapeutic target in cancer treatment.

## 2. Overview of Autophagy

Autophagy is an evolutionarily conserved intracellular recycling system and cellular self-degradation process that maintains metabolism and homeostasis. Autophagy responds to a range of cellular stresses, including nutrient deprivation, organelle damage, and abnormal protein accumulation (Figure 1) [12,13]. This autophagic process can be associated with cell death and cell survival [14,15,16]. During nutrient deprivation, autophagy is enhanced to maintain a provision of important proteins and other nutrients to serve as an energy supply, thereby increasing cell survival [17]. Recent studies reported that hypoxia can regulate autophagy, inducing processes that alleviate the oxidative stress caused by low levels of oxygen [18,19].

Under normal conditions, cells utilize basal levels of autophagy to aid in the maintenance of biological function, homeostasis, quality-control of cell contents, and elimination of old proteins and damaged organelles [1,20]. Additionally, autophagy in stem cells is related to the maintenance of their unique properties, including differentiation and self-renewal [21,22]. In cancer cells, autophagy suppresses tumorigenesis by inhibiting cancer-cell survival and inducing cell death, but it also facilitates tumorigenesis by promoting cancer-cell proliferation and tumor growth [8,9].

The mechanism of the autophagic process is controlled by a series of proteins. Mammalian target of rapamycin (mTOR) is associated with cell proliferation, stress, and cancer progression. mTOR consists of two complexes, mTORC1 and mTORC2, each of which exhibits distinct functions and localization [23,24,25]. Activated mTORC1 plays a pivotal role in the phosphorylation of autophagy-related protein (ATG) and leads to the inhibition of autophagy. When mTORC1 is inhibited under various stressful conditions, such as starvation and organelle damage, autophagy is enhanced. mTORC1 is regulated by AMP-activated protein kinase (AMPK), and inhibition of mTORC1 and increased AMPK induces the autophagic process [26,27]. However, the role of mTORC1 in autophagy is not clear [28].

When mTORC1 is inhibited, the Unc-51-like autophagy-activating kinase (ULK) complex is dephosphorylated so it becomes activated [29]. The activated ULK complex localizes to the phagophore and activates the class III PI3K [30]. Beclin-1 recruits many proteins involved in maturation and elongation of the autophagosome [31]. Elongation of autophagosome formation is regulated by ATGs. ATG5–ATG12/ATG16L complexes recruit microtubule-associated protein 1 light chain 3 (LC3) and are associated with expansion of the phagophore [32,33]. Next, LC3 drives phagophore elongation. Pro LC3 is converted to the active cytosolic isoform LC3 I by ATG4B. Next, LC3 I is converted to LC3 II by interacting with phosphatidylethanolamine (PE), ATG3, and ATG7. LC3 II is located in the inner and outer membrane of the autophagosome, enabling it to bind to degraded substrates [34,35,36]. Mature autophagosomes can fuse with lysosomes to form autolysosomes, which selectively remove proteins and damaged organelles via autophagy [37].

## 3. The Role of Autophagy in Cancer

Autophagy plays major roles in the degradation of damaged organelles and old proteins and in the maintenance of cellular homeostasis [38,39,40]. In cancer biology, autophagy plays dual roles in tumor promotion and suppression and contributes to cancer-cell development and proliferation [8,9]. Some anticancer drugs can regulate autophagy. Therefore, autophagy-regulated chemotherapy can be involved in cancer-cell survival or death [10,11]. Additionally, the regulation of autophagy contributes to the expression of tumor suppressor proteins or oncogenes. Tumor suppressor factors are negatively regulated by mTOR and AMPK, resulting in the induction of autophagy and suppression of the cancer initiation [41]. In contrast, oncogenes may be activated by mTOR, class I PI3K, and AKT, resulting in the suppression of autophagy and enhancement of cancer formation [42].

**Figure 2:**
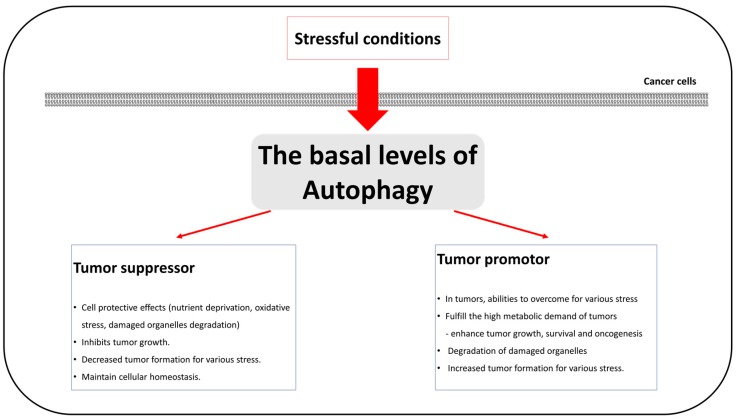
A schematic diagram of the autophagy roles of tumor promotor and suppressor in cancer cells.

Reduced and abnormal autophagy inhibits degradation of damaged components or proteins in oxidative-stressed cells, leading to the development of cancer. In addition, basal autophagy is considered a factor of cancer suppression [43,44]. The mutation of important autophagy proteins suppresses development of tumors. BIF-1 proteins that are related to BECN1 have been observed to become abnormal or absent in variety of cancer types, such as colorectal and gastric cancer [45,46,47]. UVRAG proteins are also related to BECN1 and function as autophagy regulators. The mutation of UVRAG reduces autophagy, resulting in increased cancer-cell proliferation in colorectal cancer cells [48]. On the other hand, a high basal-level of autophagy is observed in several types of RAS-activated cancer, such as pancreatic cancers [49]. Inhibition of increased autophagy in these cancers decreases cell proliferation and promotes tumor suppression [50]. Therefore, autophagy plays roles in tumor initiation and suppression, and we discuss the diverse roles of autophagy as an inducer of oncogenesis and as a tumor suppressor in following sections (Figure 2).

## 4. Autophagy as a Regulator of Tumor Suppression

The basal level of autophagy operates as a mechanism for tumor suppression via reduction of damaged cellular parts and proteins and maintenance of cellular homeostasis [51]. Previous studies have reported that the depletion of the autophagy-related gene BECN1 (encoded for Beclin 1) is observed in a variety of human breast, prostate, and ovarian cancers [44,52,53]. Beclin 1 is important in the formation of the phagophore, suggesting that Beclin 1 functions as a tumor suppressor. In cancer-cell lines and mice models, the loss of BECN1 results in a reduction of autophagy and an increase in cell proliferation, further indicating that BECN1 gene acts as a tumor suppressor [44,53,54]. In addition, several studies have shown that the level of Beclin 1 is decreased in various cancers, such as cervical squamous-cell carcinomas and hepatocellular carcinomas [55,56,57,58]. Other studies have reported that the depletion of other key autophagy genes suppresses tumor progression in cancer. A variety of proteins, including UV radiation resistance-associated gene (UVRAG) and Bax interacting factor-1 (Bif-1), which associate with BECN1 function as tumor suppressors and positively regulate autophagy [59]. The depletion of UVRAG and decrease of Bif-1 impaired autophagosome formation and autophagy, resulting in increased cancer-cell proliferation in colon, gastric, breast, and prostate cancers [45,48,60,61].

In mice with knockout of autophagic core proteins, deletion of ATG5 and ATG7 generates liver cancers from autophagy-deficient hepatocytes due to damaged mitochondria and oxidative stress [62]. Other studies have shown that the deficiency of autophagic regulators, such as ATG3, ATG5, ATG9, is associated with oncogenesis [63,64,65,66]. Mice deficient in ATG4 have been observed to have increased susceptibility to the generation of fibrosarcomas when exposed to chemical carcinogens [67]. In addition, autophagy prevents tumor generation through the regulation of reactive oxygen species (ROS). Damage to mitochondria induces excessive ROS production, resulting in promotion of carcinogenesis [68,69,70]. These results suggest that autophagy is a crucial mechanism that inhibits tumor generation, and impaired autophagy may result in oncogenesis.

## 5. Autophagy as a Regulators of Tumor Promotion

Several studies indicate that autophagy acts to promote tumor survival and growth in advanced cancers [71,72]. Tumors are exposed to extremely stressful conditions, including hypoxia and nutrient deprivation. Autophagy helps cells to overcome these stresses. Autophagy is activated in the central part of solid tumors, where cells exist under hypoxic conditions. Suppression of autophagy by deletion of Beclin 1 enhances cell death [73,74]. In addition, autophagy fulfills the high metabolic and energetic demands of proliferating tumors by recycling intracellular components to supply metabolic substrates [75,76]. In animal studies, metabolic stress is observed in autophagy-deficient cells, resulting in impaired cell survival [77]. Therefore, autophagy contributes to tumor-cell survival by enhancing stress tolerance and supplying nutrients to meet the metabolic demands of tumors, and the inhibition of autophagy or knockdown of autophagy genes can result in tumor-cell death [5,78].

Autophagy is also increased in RAS-mutated cancer cells that maintain a high basal-level of autophagy. RAS are small GTPases involved in important signal pathways for proliferation, survival, and metabolism [79,80,81]. RAS-activating mutation increases autophagy, which enhances tumor growth, survival, and oncogenesis, and is associated with the development of some deadly cancers, including lung, colon, and pancreatic [82,83,84,85]. Some studies have revealed that a high level of autophagy is observed in RAS-activating mutated cells, and cell survival is dependent on autophagy during nutrient starvation [86,87]. In addition, inhibition of autophagy-related protein enhances the accumulation of damaged mitochondria and decreases cell growth [88,89,90]. These results indicate that autophagy plays an important role in cell survival of several tumors that depend on RAS activation.

## 6. Interaction between Autophagy and Cancer Microenvironment

Many studies have shown that the cancer microenvironment is affected by autophagy in cancer cells. The cancer microenvironment consists of several factors including hypoxia, inflammation, and cytokines [91]. Autophagy supplies the demand for cellular energy and prevents cytotoxicity under stressful conditions in cancer microenvironments [92,93].

Previous studies suggested that many cancers exhibit hypoxic conditions as one of the cancer microenvironmental factors [92]. Hypoxic conditions in cancer may affect the autophagy pathway to enable cancer cells to adapt and survive under low oxygen conditions via the stress response signaling pathway, such as hypoxia-inducible factor-1 alpha (HIF-1α). Under hypoxia conditions, HIF-1α induces activation of autophagy in cancer progression [94] and regulates many of its target genes [95]. HIF-1α is also indirectly regulated by the autophagic process via changes in glucose metabolism. These processes promote glucose metabolism by HIF-1α, and a series of processes enhance autophagy [95,96]. HIF-1α-independent autophagy is associated with glucose and amino acid deprivation and activated by the induction of AMPK and inhibition of mTOR [94].

Inflammatory regulators are highly induced in the cancer microenvironment, indicating that inflammation contributes to tumorigenesis [97]. Some studies showed that inflammation triggers high levels of reactive oxygen species (ROS) in cancer cells, macrophages, and other immune cells, which secrete chemokines and cytokines, including interleukin-6, tumor necrosis factor-α, interleukin-10, and transforming growth factor-β into the cancer microenvironment [91]. These cytokines play crucial roles in inducing chronic inflammation and have anticancer effects as well as contribute to cancer progression via inflammation [98]. Inflammation is also induced by autophagy in the cancer microenvironment and cancer adjacent cells, resulting in cancer progression. Moreover, other studies demonstrated that inflammation is one factor involved in tumorigenesis and reduces autophagy activation [99]. Additionally, autophagy suppresses inflammation by inhibiting NLRP3 formation and autophagy-related protein, and ATG16L was found to modulate intestinal homeostasis and inflammation [65].

## 7. Autophagy as a Regulator of Cancer Metastasis

Cancer cells possess the ability to engage in metastasis, which is the invasion and colonization of new tissues and organs via the vascular and lymphatic systems. During metastasis, cancer cells in the origin site experience increased motility to migrate to secondary sites. In primary cancer cells, autophagy is induced by hypoxia and nutrient deprivation and protects against cell necrosis and inflammation [100,101,102]. Autophagy has demonstrated both pro-metastatic and anti-metastatic effects [100]. Autophagy acts in an anti-metastatic role via limitation of cancer necrosis and inflammation responses in early stages of cancer metastasis. In early metastasis, autophagy also reduces invasion and migration of cancer cells from origin sites. However, in advanced stages of metastasis, autophagy acts in a pro-metastatic role via promotion of cancer-cell survival and colonization in secondary sites.

Autophagy is anti-metastatic. The knockdown of autophagy-related genes, such as Beclin 1 and LC3, is inhibited proliferation, migration, and invasion, which lead to apoptosis in breast cancer [103]. A decrease in ATG5 expression, a key regulator of autophagy, reduced survival rate in 158 primary melanoma patients, and decreased ATG5 expression promotes cancer-cell proliferation and is associated with the progression of early stage cancer [104]. One study shows that blocking mTOR signaling induces autophagic cell-death and inhibits metastasis in gastric cancer cells [105].

Autophagy is also pro-metastatic. In order to undergo metastasis, cancer cells must be able to survive and proliferate in the absence of ECM, and circulation systems and dissemination to secondary sites are essential as well [106]. Cancer cell death is induced by apoptosis following the loss of ECM attachment, called anoikis. Substantial evidence indicates that autophagy enables ECM-detached cancer cells to avoid anoikis and survive [107]. One study has shown that the inhibition of autophagy reduces metastasis of hepatocellular carcinoma (HCC) in a lung metastasis model, and the inhibition of autophagy is not only reduced invasion and migration but also reduced anoikis resistance and lung metastasis of HCC cells [108]. Another study indicates that autophagy induces ECM detachment and the inhibition of β1 integrin [109].

Epithelial mesenchymal transition (EMT) is a major biological process that changes the epithelial phenotype to the mesenchymal phenotype [110]. These processes induce metastasis of cancer, leading to a lack of cell-cell adhesion, cell polarity to increase of cell motility, polarity, and invasiveness [111,112]. Additionally, EMT is important in embryonic development and affects wound healing and cancer progression. Some studies suggested that autophagy is associated with EMT in cancer. EMT-activated cancer cells have a high level of autophagy to survive under several stressful conditions during the metastatic process in cancer [113,114,115]. One study indicated that induction of autophagy by nutrient deprivation and mTOR inhibition reduced cell migration and invasion in glioblastoma cells [116]. Additionally, knockdown of autophagy-related proteins, such as Beclin 1, ATG5, and ATG7, increased the migration and invasion with EMT regulators in glioblastoma cells. Another study demonstrated that cadherin-6, a type 2 cadherin that induces EMT in embryonic development, is abnormally enhanced in cancer and associated with cancer progression [117].

## 8. Autophagy as Drug-Resistant Factor of Tumors

Several studies have revealed that the resistance of cancer cells to a variety of anticancer drugs can increase via upregulation of autophagy [118,119]. Autophagy is a protective mechanism in cancer cells undergoing anticancer therapy. Cancer chemotherapy with anticancer drugs damages dividing cells and interrupts cancer-cell division. Chemotherapy is a common treatment strategy for cancer therapy, but the success rate of chemotherapy is often limited because of the development of chemoresistance. In particular, induction of protective autophagy is a major challenge in cancer therapy. 5-Fluorouracil (5FU) is one anticancer drug used in solid cancers, such as breast, pancreatic, and colorectal cancer [120]. 5FU inhibits thymidylate synthetase and then leads to the inhibition of DNA synthesis [121]. However, the efficacy of treatment with 5FU is restricted, because protective autophagy is induced, resulting in chemoresistance in various cancer cells. The mechanism of protective autophagy is induced by beclin-1 expression, followed by conversion of LC3I to LC3II. Then, JNK-mediated protective autophagy and an increase of BCL2 increases autophagic flux and induces chemoresistance [121,122,123].

In addition, cisplatin is a primary treatment drug in many solid cancers, and its anticancer effects are induced by the generation of DNA damage and mitochondrial apoptosis [124]. However, the efficacy of treatment with cisplatin is restricted by the development of chemoresistance [125]. As the mechanism of cisplatin-mediated resistance, autophagy contributed to drug resistance in ovarian cancer via the modulation of ERK pathway and overexpression of Beclin 1 [126,127]. Another study has shown that cisplatin treatment promotes protective autophagy via the upregulation of Beclin 1, conversion of LC3 proteins, and increase of ATG7 expression in esophageal cancer [128,129]. Furthermore, cisplatin treatment combined with the inhibition of autophagy significantly increased cytotoxicity in esophageal cancer [130]. Chemoresistance to various chemotherapeutic agents is induced by protective autophagy in many cancers. Therefore, the efficacy of chemotherapeutic agents in many cancers is restricted by the induction of unexpected protective autophagy, and chemoresistance could be overcome via investigation of the autophagy pathway and the proper regulation of autophagy levels.

Autophagy contributes to the application of immunity in anticancer therapy. Immune effects in anticancer therapy occur through several steps. Cancer neoantigen is released into the surrounding environment and presented to T cells, and then cancer cells are killed through T cell-mediated cytotoxicity [131]. Other immune related cells are also induced and eliminate cancer cells presenting neoantigen through the generation of interferon-γ and perforin. Autophagy is associated with the survival and death mechanisms in many cell types. Inhibition of autophagy-related proteins, including Beclin 1 and ATG7, impairs the survival of T cells by damaging ER homeostasis, causing mitochondrial dysfunction, and increasing ROS [132,133]. Autophagy is associated with T-cell survival. However, hypoxia-mediated autophagy leads to resistance of T-cell cytotoxicity via activation of STAT3. One study showed that the inhibition of autophagy by silencing of Beclin 1 and ATG5 decreased hypoxia-induced activation of STAT3 and restored cancer cell sensitivity to T-cell cytotoxicity [134]. Therefore, abnormal activation of STAT3 reduced the sensitivity of the immune response.

## 9. The Role of Autophagy in Cancer Stem-Cells

Cancer stem-cells (CSC) are a small subpopulation of cancer cells that have the abilities of self-renewal and differentiation and contribute to tumor initiation, chemoresistance, and metastasis [135,136]. Several studies have investigated the mechanism underlying maintenance of stemness maintenance in these cells, and autophagy may play an important role in this process [137,138]. A previous study has shown that autophagy regulates homeostasis of CSCs [139].

In glioma stem cells, the inhibition of autophagy suppresses differentiation, whereas enhancement of autophagy promotes differentiation [140]. A study has revealed that the decrease of LC3B-II and Beclin-1 are related to the development of astrocytic tumors [58]. However, one study indicates that autophagic cell death is observed in glioma stem cells. The suppression of autophagy by cilengitide, an integrin antagonist, attenuates cytotoxicity [141]. Thus, it is unclear whether autophagy regulates stemness in glioma stem cells.

In breast cancer stem-cells, a previous study has shown that CSCs contribute to the recurrence of cancer and metastasis [142]. Autophagy is observed to positively modulate mesenchymal-like phenotypes in breast cancer stem-cells. Silencing of two autophagy-related proteins, LC3B and ATG12, or treatment with autophagy inhibitors directly reduces cancer stem-cell-like phenotypes [143]. Several studies demonstrate that autophagy is associated with protective effects against various cellular stresses in breast CSCs [144,145]. Other studies have confirmed that the inhibition of autophagy promotes the sensitization of cancer cells to anticancer therapy [146,147]. Also, under hypoxia and metabolic stress, necrosis happens in carcinoma and can induce inflammation and enhance the recruits of inflammatory cell, and then promotes pro-metastasis [148,149]. Autophagy inhibits necrosis and inflammation at cancer sites by producing cellular energy and metabolic precursors, followed by the suppression of metastasis. EMT is another important factor in cancer metastasis. Some regulators of EMT can promote EMT through the loss of cell–cell adhesion and associations with autophagy. One study suggested that defects of autophagy enhance EMT by stabilizing TWIST1 [150].

**Figure 3:**
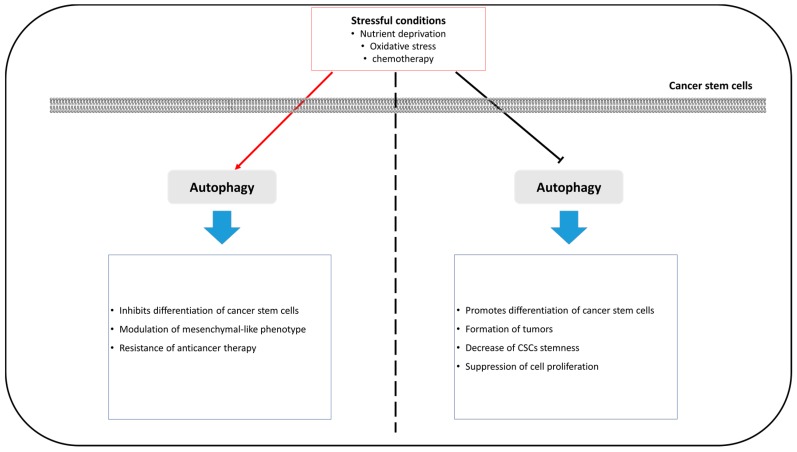
A schematic diagram of the autophagy roles in cancer stem-cells.

In colorectal cancer stem-cells, the maintenance of pluripotency in CSCs requires complex responses, such as energy metabolism, during a variety of stressful conditions. Autophagy is one of the responses to stressful conditions and maintains pluripotency in colorectal CSCs. Sharif et al. have demonstrated that the knockdown of autophagy-related proteins, ATG5 and ATG7, inhibits autophagy and reduces stemness markers, such as Oct4, SOX2, and Nanog. As a result, reduced autophagy leads to suppressed cell-proliferation and enhanced cell-senescence in colorectal CSCs [139]. Another study has indicated that induction of autophagy by overexpressing prion protein induces resistance to anticancer reagents and sustains stemness in colorectal CSCs [151]. These results indicate that autophagy is a core regulator of pluripotency maintenance and drug resistance in CSCs (Figure 3).

## 10. Targeting Autophagy in Cancer Therapy

Many studies have indicated that autophagy functions as both a tumor suppressor and promoter [152]. The modulation of autophagy is a promising potential strategy to enhance cancer therapy. A previous study has identified drugs targeting all steps of the autophagic processes, from the initiation of the autophagosome to the degradation step [153]. In addition, many studies have confirmed that autophagy plays crucial roles in anticancer therapy, including the acquisition of resistance for anticancer therapy [154,155,156,157]. Autophagy enhanced by chemotherapy decreases cell death, increases cancer-cell survival, and is associated with drug resistance in cancer. A previous study found that autophagy facilitates cancer-cell survival and drug resistance to anticancer reagents and maintains stem cell-like properties in hepatocellular carcinoma [158]. Another study indicates that the suppression of autophagy leads to promotion of apoptosis and therapeutic effects of anticancer therapy [159]. In addition, use of the autophagy inhibitor chloroquine can enhance apoptosis and the therapeutic effects of photososan-II-mediated photodynamic therapy (PS-PDT) in colorectal cancer cells [160]. Much evidence supports autophagy modulation as a promising and potential therapeutic target.

Some autophagy regulators, such as rapamycin, rapamycin water-soluble derivatives (temsirolimus and everolimus), chloroquine (CQ: antimalarial agent), and hydroxychloroquine (HCQ: CQ derivative), are used in cancer therapy. Temsirolimus and everolimus, which inhibit mTORC1 and induce autophagy, are approved by the Food and Drug Administration (FDA) for cancer therapy. Everolimus is used to treat progressive neuroendocrine tumors of pancreatic origin (PNET) and breast cancer combined with exemestance [161]. In addition, temsirolimus is used to treat relapsed or refractory mantle-cell lymphoma in the European Union [162,163,164], while rapamycin is used in coronary stents and rare pulmonary diseases [165,166].

Both CQ and HCQ, which have previously been used to prevent and treat malaria, are lysosomal inhibitors and directly inhibited autophagy via alteration of lysosomal pH, inhibition of autophagic degradation, and accumulation of autophagosomes [167,168,169]. Preclinical studies have shown that CQ or HCQ can suppress cancer-cell growth via the inhibition of autophagy in bladder cancer and pancreatic adenocarcinoma [170,171]. In addition, some studies have indicated that these reagents enhance the therapeutic effects of chemotherapy through the inhibition of autophagy-mediated resistance to cancer therapy [123,172,173].

Moreover, Lys05 is a water-soluble analog of HCQ and was developed as a new lysosometropic agent [5]. It functions at a low dose and increases the pH of the lysosome, resulting in autophagy inhibition [174]. Lys05 showed higher anticancer effects than HCQ in vitro and in vivo in melanoma and colon cancer xenograft models [175]. Additionally, Lys05 combined with a BRAF inhibitor effectively inhibited cancer in vivo [176]. Therefore, the development of autophagy-specific inhibitors is a new therapeutic strategy for anticancer therapy.

Other autophagy-related drugs have been developed for anticancer therapy. Spautin-1 inhibits autophagy and leads to the induction of proteasomal removal of class III PI3K kinase complexes [177]. The pro-apoptotic effect of Spautin-1 is related to GSK3β and affects a crucial downstream effector of PI3K/Akt [178]. Therefore, Spautin-1 is a potential therapeutic agent for anticancer therapy. Additionally, SAR405 is a kinase inhibitor of Vps18 and Vps34 and impairs lysosomal function [179], as well as influences the interaction between the late endosome and lysosome [180]. SAR405 combined with everolimus enhances the inhibition of cancer proliferation in renal cancer cell lines [179]. These results indicate that SAR405 has anticancer therapeutic effects as a Vps34 inhibitor.

## 11. Conclusions

Autophagy is regulated by complex intracellular processes under stressful conditions, including nutrient deprivation, the presence of damaged organelles, and anticancer therapy. Many studies have found that autophagy plays dual roles in cell survival and cell death in the context of tumor initiation and development. Studies on autophagy-deficient mouse models indicate that the basal levels of autophagy can suppress tumor formation at the initiation of tumor development. However, autophagy promotes cancer progression in many cancers. Many studies demonstrate that autophagy supplies sufficient nutrients that enable cancer cell growth. However, some studies indicated that autophagy also suppresses tumor growth, initiation, and development. Some autophagy-related genes plays roles in non-autophagy functions in addition to autophagy-related functions [181]. FIP200, an autophagy-related protein, interacts with ATG 13 and induces autophagy. Although mutations in FIP200 induce autophagy, FIP200 plays a role in embryogenesis via the protection of apoptosis [181]. Another study revealed that the loss of autophagy-related genes increased the aggressive development of HER2-positive breast cancer [182]. Increased autophagy induces the interaction of Beclin 1 and HER2, resulting in increased tumorigenesis. In BECN1 mutant mice, augmentation of autophagy did not induce HER2-mediated tumorigenesis. Therefore, in order to fully apply the properties of autophagy in cancer therapy, additional study on its role in the disease within a variety of biological fields is necessary.

CSCs have the ability to self-renew and form cancers, and the presence of CSCs contributes to resistance to anticancer therapy, recurrence, and metastasis. Autophagy mitigates several stressful conditions and promotes resistance to anticancer therapy. Moreover, autophagy regulates maintenance of pluripotency in CSCs and leads to failure of anticancer therapy. Therefore, autophagy is a potential therapeutic target to address the resistance of anticancer therapy, recurrence, and metastasis. However, additional study is required before CSCs can be treated by modulating autophagy. Regulation of autophagy using autophagy modulators alone has not improved the therapeutic effects of anticancer reagents; on the contrary, it has supplied nutrients for cancer cells. Thus, clinical trials that target autophagy through a combination of autophagy modulations and anticancer reagents are necessary to investigate autophagy as a potentially effective therapeutic strategy in anticancer therapy.
